# Maximizing Satisfaction in Orthopedic Outpatient Clinics: Evidence From Ireland

**DOI:** 10.7759/cureus.63104

**Published:** 2024-06-25

**Authors:** Abobaker Younis, Mehad Elmubarak, Hussam Elkhwad, MN Baig, Mohamed Saeed, Ayman Omer

**Affiliations:** 1 Orthopaedics and Traumatology, University Hospital Galway, Galway, IRL; 2 Orthopaedics, University Hospital Galway, Galway, IRL

**Keywords:** cross-sectional study, orthopedic clinic, galway, ireland, patients’ satisfaction

## Abstract

Background

Patient satisfaction is a critical metric in healthcare, reflecting the quality of care provided and influencing clinical outcomes and healthcare utilization. In orthopedic outpatient clinics, patient satisfaction affects patient adherence to treatment plans and overall health outcomes. This study aims to identify and analyze key factors influencing patient satisfaction in orthopedic outpatient clinics.

Methodology

This cross-sectional study was conducted from April to May 2024 across 10 orthopedic outpatient clinics. In this study, family members were included as respondents to the Patient Satisfaction Questionnaire (PSQ) when patients were unable to complete the survey due to age, cognitive impairment, or physical disabilities. This approach was adopted to ensure that the experiences of all patients, particularly minors, elderly individuals, and those with disabilities, were accurately captured. The PSQ assessed various aspects of patient satisfaction, including communication, treatment plans, addressing concerns, clinic environment, and overall satisfaction. Quantitative data were analyzed using SPSS version 27.0 (IBM Corp., Armonk, NY, USA).

Results

The study included 172 respondents. High levels of overall satisfaction were reported, with 142 (82.6%) respondents very satisfied and 28 (16.2%) somewhat satisfied. Significant associations were found between overall satisfaction and several factors, namely, effective communication, thorough explanation of treatment plans, addressing patient concerns, and a clean, comfortable clinic environment. Shorter waiting times were also associated with higher satisfaction. Regression analysis revealed that staff rating and the thoroughness of treatment plans were significant predictors of overall satisfaction.

Conclusions

Effective communication, thorough treatment explanations, addressing patient concerns, and maintaining a clean clinic environment are key determinants of patient satisfaction in orthopedic outpatient clinics. Reducing waiting times and investing in staff training on communication and empathetic care can further enhance patient satisfaction. These findings provide valuable insights for healthcare providers and administrators aiming to improve patient experiences in orthopedic outpatient settings. Further research is recommended to explore these relationships in diverse settings and develop targeted interventions.

## Introduction

Patient satisfaction is a crucial metric in healthcare, serving as an indicator of the quality of care provided and directly influencing clinical outcomes and healthcare utilization. In the context of orthopedic outpatient clinics, patient satisfaction not only reflects the quality of clinical interactions but also affects patient adherence to treatment plans and overall health outcomes [1]. Understanding the factors that influence patient satisfaction in these settings is essential for developing strategies to improve patient experiences and clinical outcomes.

Several studies have highlighted the importance of minimizing wait times to enhance patient satisfaction. Kreitz et al. found that shorter wait times in orthopedic clinics significantly improved patient satisfaction scores, emphasizing the need for efficient clinic management [2]. However, Patterson et al. noted that while time with the provider had a positive impact on patient satisfaction, the overall clinic wait time did not significantly affect satisfaction among orthopedic patients [3]. This suggests that the quality of interaction with healthcare providers may outweigh the inconvenience of waiting.

Effective communication between healthcare providers and patients is another critical determinant of patient satisfaction. Stephens et al. demonstrated that clear and thorough communication during consultations significantly enhances patient satisfaction in orthopedic outpatient settings [4]. Similarly, Waters et al. validated a patient questionnaire that assessed satisfaction with orthopedic consultations, further underscoring the role of effective communication in improving patient experiences [5].

The involvement of advanced practice providers, such as physician assistants, has also been associated with higher patient satisfaction in orthopedic outpatient clinics. Sharabianlou et al. reported that the presence of a physician assistant during outpatient visits was linked to increased patient satisfaction, suggesting that these providers can play a vital role in patient care [6].

Moreover, factors such as the physical environment of the clinic and the perceived competence and empathy of the healthcare staff are also significant contributors to patient satisfaction. Li et al. conducted a systematic review identifying various factors associated with outpatient satisfaction in tertiary hospitals, including the cleanliness and comfort of the clinic environment [7]. In orthopedic settings, the ability of the staff to address patient concerns empathetically and competently is paramount, as highlighted by numerous studies [8].

The purpose of this study is to identify and analyze the key factors influencing patient satisfaction in orthopedic outpatient clinics. By understanding these factors, healthcare providers can implement targeted interventions to enhance patient experiences, improve clinical outcomes, and increase overall satisfaction with orthopedic care.

## Materials and methods

This study employed a cross-sectional survey design to assess patient satisfaction in orthopedic outpatient clinics. The survey was conducted over one month from April 2024 to May 2024 across 10 trauma clinics in a major metropolitan area.

Participants included patients who attended the orthopedic outpatient clinics during the study period. Inclusion criteria were patients who received care in the clinic. In this study, family members were included as respondents to the Patient Satisfaction Questionnaire (PSQ) when patients were unable to complete the survey due to age, cognitive impairment, or physical disabilities. This approach was adopted to ensure that the experiences of all patients, particularly minors, elderly individuals, and those with disabilities, were accurately captured. Including family members as proxies provides a comprehensive view of patient satisfaction and reflects the realities of the patient population served in orthopedic outpatient clinics.

The primary data collection tool was a structured PSQ developed based on existing validated instruments [1,2,5]. The PSQ comprised the following four sections: demographic information, appointment details, experience with service, and overall satisfaction. Each section contained multiple-choice and Likert-scale questions designed to capture various aspects of patient satisfaction.

The survey instrument included the following sections: (1) Demographic information: age, gender, and outcome of the appointment (discharged or booked for another appointment). (2) Appointment details: waiting time past the appointment time, and convenience of access to required departments. (3) Experience with service: clarity of communication from healthcare providers, thoroughness of treatment explanation, addressing of concerns, and clinic environment (cleanliness and comfort). (4) Overall satisfaction: overall satisfaction with the visit, the importance of seeing a consultant, and open-ended questions about the most liked aspects and areas for improvement.

Patients were invited to participate in the survey immediately following their clinic visit. Trained research assistants approached patients in the waiting area, provided an explanation of the study, and obtained informed consent. Surveys were administered in paper format and took approximately 10-15 minutes to complete. Completed surveys were collected by the research assistants and entered into a secure database for analysis.

Verbal informed consent was obtained from all participants before survey administration. Participant confidentiality was maintained by assigning unique identifier codes to each survey and storing data in a password-protected database. Only aggregated data were reported to ensure anonymity.

Quantitative data were analyzed using SPSS version 27 (IBM Corp., Armonk, NY, USA). Descriptive statistics (means, standard deviations, frequencies, and percentages) were used to summarize demographic characteristics and survey responses. Inferential statistics, including chi-square tests and t-tests, were employed to examine associations between demographic variables and patient satisfaction scores. A p-value <0.05 was considered statistically significant.

## Results

A total of 172 respondents completed the PSQ, including proxies such as parents, family members, or caregivers to capture the experience of significant service receivers. Among them, 114 (66.3%) were patients themselves, 56 (32.6%) were family members, and two (1.2%) were caregivers. The respondents’ ages ranged from 13 to 92 years, with a mean age of 45.77 years (SD = 17.70). The gender distribution included 64 (37.2%) males and 108 (62.8%) females. Regarding the appointment outcome, 78 (45.3%) respondents were discharged, while 94 (54.7%) were booked for another appointment (Table 1).

**Table 1:** Demographic characteristics. The table shows the demographic distribution of the respondents, including gender, age, the role of the person filling out the questionnaire, and the outcome of their visit. Mean age is presented with its standard deviation (SD).

Demographic	n	%
Gender		
Male	64	37.2%
Female	108	62.8%
Age		
Mean (SD)	45.77 (17.70)	-
Who filled the questionnaire		
The patient	114	66.3%
Family member	56	32.6%
Caregiver	2	1.2%
Outcome of visit		
Discharged	78	45.3%
Booked for another appointment	94	54.7%

The average waiting time past the appointment time was 32.29 minutes (SD = 30.23), with reported times ranging from 0 to 120 minutes. A significant majority, 152 (88.4%), reported that access to the required department was convenient. Regarding service experience, 118 (68.6%) rated communication from healthcare providers as excellent, 50 (29.1%) as good, and four (2.3%) as average. For the explanation of treatment plans, 136 (79.1%) felt their treatment plans were explained very thoroughly, 26 (15.1%) somewhat thoroughly, and 10 (5.8%) acceptably. Regarding addressing concerns, 146 (84.9%) felt their concerns were addressed very well, 22 (12.8%) adequately, and four (2.3%) acceptably. The clinic environment was rated as excellent by 122 (70.9%), good by 46 (26.7%), and average by four (2.3%). Additionally, 110 (64.0%) respondents saw a consultant during their visit (Table 2).

**Table 2:** Descriptive statistics for service experience. The table outlines the respondents’ service experience, including their ratings of communication, treatment plans, addressing concerns, environment, and whether they saw a consultant during their visit.

Aspect of service	Rating category	n	%
Eases of access	Yes	152	88.4%
	No	20	11.6%
Communication	Excellent	118	68.6%
	Good	50	29.1%
	Average	4	2.3%
Treatment plans	Very thoroughly	136	79.1%
	Somewhat thoroughly	26	15.1%
	Acceptably	10	5.8%
Addressing concerns	Very well	146	84.9%
	Adequately	22	12.8%
	Acceptably	4	2.3%
Environment	Excellent	122	70.9%
	Good	46	26.7%
	Average	4	2.3%
Seen consultant today	Yes	110	64.0%
	No	62	36.0%
Importance of seeing a consultant	Very important	96	55.8%
	Somewhat important	48	27.9%
	Neither important nor unimportant	28	16.3%

**Figure 1:**
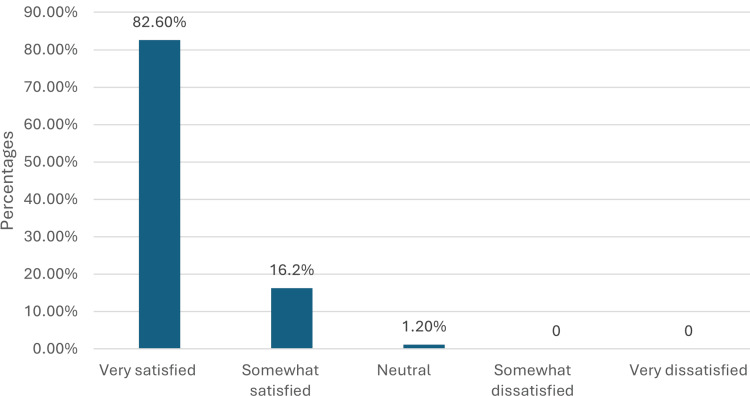
Distribution of overall satisfaction ratings. The bar chart illustrates the distribution of overall satisfaction ratings among respondents. The majority of respondents reported being very satisfied, followed by somewhat satisfied, with a very small percentage being neutral or dissatisfied.

Staff ratings were predominantly high, with 146 (84.9%) rating the staff as excellent, 24 (14.0%) as good, and two (1.2%) as average. Overall satisfaction was very high, with 142 (82.6%) respondents being very satisfied, 28 (16.3%) somewhat satisfied, and two (1.2%) neutral (Figure 1). Regarding the importance of seeing a consultant, 96 (55.8%) felt it was very important, 48 (27.9%) somewhat important, and 28 (16.3%) neither important nor unimportant.

A significant association was found between overall satisfaction and age (chi-square = 162.930, p < 0.001), with younger patients tending to report higher satisfaction levels. However, there was no significant association between overall satisfaction and gender (chi-square = 3.423, p = 0.181), nor between overall satisfaction and the outcome of the appointment (chi-square = 1.803, p = 0.406). There was a significant association between overall satisfaction and waiting time (chi-square = 51.513, p = 0.028), with shorter waiting times associated with higher satisfaction. No significant association was found between overall satisfaction and ease of access (chi-square = 0.474, p = 9.789) (Table 3).

**Table 3:** Chi-square test results for overall satisfaction. The table presents the chi-square test results for associations between overall satisfaction and various demographic and service-related variables. A significant p-value (<0.05) indicates a strong association.

Variable	Chi-square	p-value
Age	162.930	<0.001
Gender	3.423	0.181
Outcome	1.803	0.406
Waiting time	51.513	0.028
Ease of access	0.474	0.789
Communication	31.506	<0.001
Treatment plans	57.770	<0.001
Address concerns	31.879	<0.001
Environment	20.090	<0.001
Seen a consultant	1.149	0.563
Staff rating	13.611	0.009
Importance of seeing a consultant	14.594	0.006

Significant associations were found between overall satisfaction and several aspects of the service experience: communication (chi-square = 31.506, p < 0.001), thoroughness of treatment plans (chi-square = 57.770, p < 0.001), addressing concerns (chi-square = 31.879, p < 0.001), and the clinic environment (chi-square = 20.090, p < 0.001). There was no significant association between overall satisfaction and seeing a consultant (chi-square = 1.149, p = 0.563), but there was a significant association between overall satisfaction and staff rating (chi-square = 13.611, p = 0.009) and the importance of seeing a consultant (chi-square = 14.594, p = 0.006).

Correlation analysis revealed significant positive correlations between overall satisfaction and communication (r = 0.300, p < 0.001), thoroughness of treatment plans (r = 0.484, p < 0.001), addressing concerns (r = 0.332, p < 0.001), environment (r = 0.325, p < 0.001), and staff rating (r = 0.167, p = 0.029) (Table 4).

**Table 4:** Correlation analysis for overall satisfaction. The table shows the correlation coefficients (r) and p-values for the relationship between overall satisfaction and various service experience variables. Significant correlations (p < 0.05) indicate a strong relationship.

Variable	Correlation (r)	P-value
Communication	0.300	<0.001
Treatment plans	0.484	<0.001
Address concerns	0.332	<0.001
Environment	0.325	<0.001
Staff rating	0.167	0.029

## Discussion

The results of this study provide valuable insights into the factors influencing patient satisfaction in orthopedic outpatient clinics. The high levels of overall satisfaction observed among respondents indicate that, generally, patients have a positive perception of the care they receive. This discussion will compare our findings with existing literature, emphasizing key factors such as communication, clinic environment, waiting time, staff competence, and treatment explanations.

Effective communication between healthcare providers and patients was significantly associated with higher overall satisfaction in our study. This finding corroborates the work of Stephens et al. (2020) and Waters et al. (2021), who demonstrated that clear and thorough communication during consultations significantly enhances patient satisfaction in orthopedic outpatient settings [4,5]. Similar conclusions were drawn by Donnally et al. (2019) and Zhang et al. (2017), highlighting the critical role of effective communication in improving patient experiences in orthopedic clinics [9,10]. Our study adds to this body of evidence by quantifying the positive impact of communication on patient satisfaction, emphasizing the need for healthcare providers to prioritize clear and empathetic communication.

The clinic environment was another significant factor influencing patient satisfaction in our study. A clean and comfortable environment was positively associated with higher satisfaction ratings. This finding is supported by previous research, such as the systematic review by Li et al. (2020), which identified the cleanliness and comfort of the clinic environment as significant contributors to patient satisfaction [7]. Redding et al. (2022) similarly found that a positive clinic environment significantly impacts patient satisfaction in pediatric otolaryngology clinics [11]. Etier et al. (2016) also emphasized the importance of the clinic environment in orthopedic spine clinics [12]. These studies, along with our findings, underscore the importance of maintaining a welcoming and hygienic clinic setting to enhance patient satisfaction.

Our study found that shorter waiting times were associated with higher patient satisfaction, consistent with the findings of Kreitz et al. (2016) and Rane et al. (2019) [2,13]. These studies demonstrated that efficient clinic management and reduced waiting times are crucial for improving patient experiences. However, Patterson et al. (2017) noted that overall clinic wait time did not significantly affect satisfaction among orthopedic patients, suggesting that the quality of interaction with healthcare providers may outweigh the inconvenience of waiting [3]. This discrepancy highlights the need for further research to understand the nuances of how waiting time influences satisfaction in different contexts.

The competence and empathy of healthcare staff were also significantly associated with overall satisfaction in our study. This finding aligns with previous studies by Sharabianlou et al. (2022) and Singleton et al. (2021), which emphasized the critical role of staff competence and empathetic interactions in enhancing patient satisfaction [6,14]. Bak Bødskov et al. (2022) reported similar findings in orthopedic outpatient shoulder clinics, highlighting the importance of competent and caring staff in improving patient experiences [15]. Our study reinforces these conclusions, suggesting that continuous training and development of healthcare staff are essential for maintaining high levels of patient satisfaction.

Thorough explanations of treatment plans emerged as a critical determinant of patient satisfaction in our study. This is consistent with the findings of Sharabianlou et al. (2022) and Abtahi et al. (2015), who reported that detailed explanations of treatment options were associated with increased patient satisfaction [6,16]. Waters et al. (2016) also found that clear explanations during orthopedic consultations significantly impacted patient satisfaction [17]. Rabah et al. (2021) identified key drivers of patient satisfaction with spine surgeons, including effective communication and thoroughness of care [18]. These findings, along with ours, highlight the importance of ensuring that patients fully understand their treatment plans, which can enhance their trust in healthcare providers and adherence to medical advice.

This study has some limitations that should be considered when interpreting the findings. The cross-sectional design limits the ability to infer causality between the identified factors and patient satisfaction. Additionally, the study was conducted in a specific geographic area, which may limit the generalizability of the findings to other settings. The relatively small sample size of 172 respondents may not fully capture the diversity of patient experiences. Furthermore, respondent-related bias may exist, as responses from family members and caregivers might reflect their perspectives rather than those of the patients themselves. Future research should consider longitudinal designs to explore the causal relationships between these factors and patient satisfaction. Moreover, studies conducted in diverse geographic locations and clinic settings could help validate the findings and enhance their generalizability.

## Conclusions

This study identifies several key factors that influence patient satisfaction in orthopedic outpatient clinics, including effective communication, thoroughness of treatment plans, addressing patient concerns, and maintaining a clean and comfortable clinic environment. The findings underscore the importance of these factors in enhancing patient satisfaction and provide valuable insights for healthcare providers and administrators aiming to improve patient experiences in orthopedic outpatient settings. Further research is needed to explore the nuances of these relationships and develop targeted interventions to enhance patient satisfaction.
